# Helminth eggs from early cretaceous faeces

**DOI:** 10.1038/s41598-020-75757-4

**Published:** 2020-10-30

**Authors:** Sandra Barrios-de Pedro, Antonio Osuna, Ángela D. Buscalioni

**Affiliations:** 1 Unidad de Paleontología and Centro para la Integración en Paleobiología (CIPb), Departamento de Biología, Edificio de Biología, Universidad Autónoma de Madrid, Calle Darwin 2, Cantoblanco, 28049 Madrid, Spain; 2 Departamento de Parasitología, Facultad de Ciencias, Universidad de Granada, Granada, 18071 Granada, Spain; 3 Instituto Universitario de Biotecnología, Granada, 18071 Granada, Spain

**Keywords:** Evolution, Ecology

## Abstract

The exceptional fossil site of Las Hoyas (upper Barremian, Cuenca, Spain) yields abundant small to medium vertebrate coprolites, hindering the search for parasites. We studied the contents of 29 coprolites that were previously classified into distinct morphotypes. Several parasitic eggs were retrieved from two of these coprolites, confirming the second record of digenea trematode eggs and nematode (ascaridid) eggs from an Early Cretaceous locality. The cylindrical coprolite containing anisakid eggs was likely produced by a crocodylomorph as the parasite host, whereas the bump-headed lace coprolite indicates the role of a fish as an intermediary or definitive host of the trematodes and ascaridids. These trace and body fossils show that the Las Hoyas 126–129 Ma lacustrine ecosystem documents the early connection between basal Gonorynchiformes fish and digenetic trematodes.

## Introduction

The identification of parasitic material in coprolites (fossil faeces) remains a challenge, but can provide substantial information on the habitat of parasitic hosts and the feeding habits of infected animals. However, the identification of parasites in fossil faeces is complex since such parasites must be accurately located to avoid their destruction. Furthermore, these parasites need to be preserved and they must be recognized according to their modern analogues. Fossil parasites are mostly in eggs and cysts whose preservation depends on the presence of layers that degrade slowly and conditions that favour the integrity of the faecal mass. For example, preservation of coprolites bearing parasite remains would have been aided by quick drying to prevent the dispersion of parasitic elements, occurring in an anaerobic environment to slow the destruction of the faecal mass by bacteria and fungi, and quick covering by microbial biofilms and mats^1^.

Despite the difficulty in studying fossil parasites, the evolution of helminths constitutes an active and integrative research area that incorporates parasite-body fossils and other information recorded in coprolites since the Palaeozoic^2^. Well-known records correspond mostly to eggs retrieved in coprolites, such as those found in a shark coprolite from the Permian in Brazil^3^; a nematode in a cynodont coprolite from the Triassic in Brazil^4^; Ascaridae nematodes in coprolites of cynodonts and crocodiles from the Triassic and Cretaceous in Brazil^5,6^; and a dinosaur faecal mass from the Cretaceous in Bernissart (Belgium)^7^. Holocene fossil parasites are not uncommon, and cestode eggs in rodent coprolites, as well as helminth and coccidian oocysts in deer, fox, or feline coprolites, have been described from several archaeological sites in Patagonia (Argentina)^8–11^.

In our research programme on coprolites from Las Hoyas, we searched parasitic structures^12^. Las Hoyas is a well-known exceptional upper Barremian deposit made famous by the discovery of articulated and soft-bodied animals with preserved soft- tissues^13^. This soft-tissue preservation is linked to the presence of microbial mats, which would have played a crucial role in maintaining the integrity of the organism bodies and the biological rests therein produced^14–16^. According to the palaeogeographic, sedimentological, and palaeontological data, this Barremian locality was an inland freshwater ecosystem with subtropical seasonality and no marine influence^17–19^. In the Las Hoyas ecosystem, obligate aquatic organisms (e.g., fish, annelids, molluscs, crustaceans, and some insects) are more common than vertebrates with aquatic-dependent life cycles (salamanders, frogs, crocodiles, and turtles), and terrestrial animals (some insects, diplopods, albanerpetontids, squamates, dinosaurs, and birds)^20^.

Coprolites are one of the most common fossils in this locality. The difficulty in finding parasites in these coprolites is related to the sizes and diameters of coprolites, which are on average 15 mm in length and 5 mm in diameter^12^. Here, we present a protocol prepared by the Molecular Parasitology Department from the University of Granada (Spain) that removes minerals without dissolving the parasitic remains (see “Methods” section). The Las Hoyas coprolites preserve valuable information on the presence of parasitic groups in the lacustrine Mesozoic ecosystem, and on the degree of complexity in their life cycles. Importantly, the Las Hoyas biota include “worms”^21^ attributed to several annelids of Oligochaeta (Tubificidae and several undetermined shapes) and Nematoda (Mermithidae), which include parasitic species. Trace fossils produced by invertebrates have also been recorded in the Las Hoyas deposit^22,23^. Therefore, we include relevant aspects related to the palaeoecology of the ecosystem based on a study of coprolites.

## Coprolites and parasites in Las Hoyas

**Figure 1:**
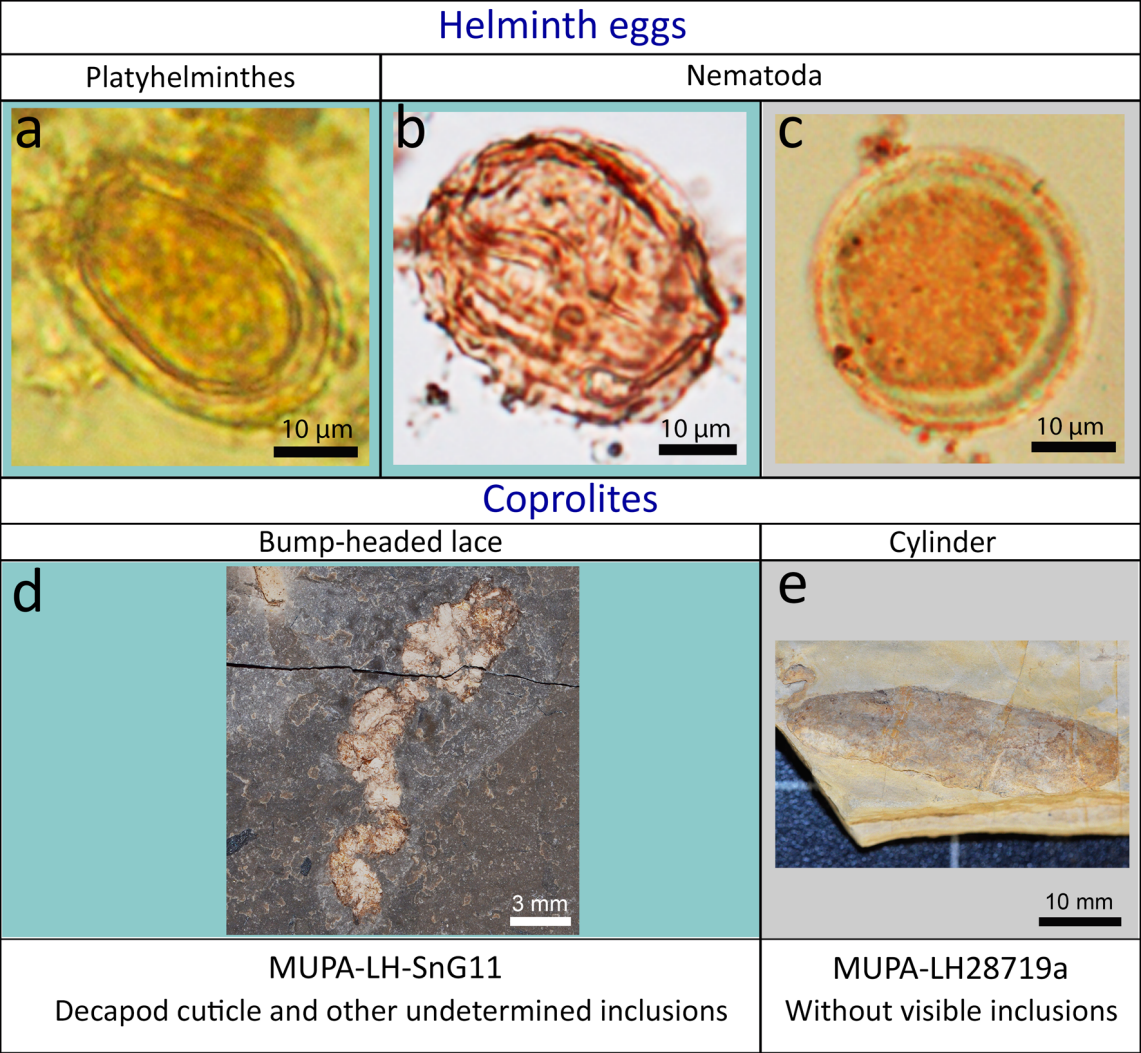
Parasite egg-like structures found in the Las Hoyas coprolites: (**A**) Platyhelminth egg-like, MUPA-LH-SnG11-Tr; (**B**) Nematode egg-like, MUPA-LH-SnG11-As; (**C**) Nematode egg-like (probably an anisakid), MUPA-LH28719a-As. Macrophotographs of the coprolites where the parasite eggs were found: (**D**) Bump-headed lace coprolite, MUPA-LH-SnG11; (**E**) Cylinder coprolite, MUPA-LH28719a. The blue background indicates the parasite eggs found in the bump-headed lace coprolite, and the grey background indicates the parasite egg found in the cylinder coprolite.

Coprolites are one of the most common fossils in Las Hoyas. A great variety of coprolite shapes, from small to medium, has been previously described and analysed, comprising a total of twelve coprolite morphotypes^12,24^. We have used a morphological classification to test differences within the locality and to more precisely associate the coprolite shape with the recorded producers. The coprolite sample herein analysed contains 29 specimens with ten different shapes, including broken coprolites (see Supplementary Information). Three egg-like structures of intestinal parasites were found in two specimens: in the bump-headed lace coprolite MUPA-LH-SnG11, and in the cylinder MUPA-LH28719a (see Fig. 1).

Based on their shapes and sizes, the chemical and stable isotope analyses performed on them, the description of their coprofabrics, and a valuation of the taphonomic alterations of their inclusions, it was concluded that the Las Hoyas coprolites were produced by vertebrates, mostly feeding on aquatic food sources^12^. Fishes and crustaceans (shrimp and decapods) were the preferred prey^24,25^. Lace-shaped coprolites (i.e., thin lace, straight lace, and bump-headed lace) accounted for the highest percentage of the whole coprolite sample (47%), and they were produced by fishes. In fact, fishes are the most abundant and diverse group at Las Hoyas, including Actinopterygii, Holostei, Teleostei, and Coelacanthiformes^26,27^. The bump-headed lace coprolites were confidently attributed to teleost fishes, because of their diameter, length, shape, and type and density of their inclusions, but small amiiforms have also been discussed as potential producers^12,25^. However, cylinder coprolites are produced by a great diversity of animals, such as lizards, turtles, crocodiles, and (likely) coelacanths. These types of coprolites are characterized by a matrix generally flacking inclusions that, together with the coprolite diameter, is usually interpreted as being produced by a reptile, likely an archosaur crocodylomorph^12^. The parasites found here were identified based on our broad teaching and research experience, as well as the use of parasitology atlases, publications, and books^28–31^.

The parasites found in the Las Hoyas coprolites (Fig. 1A–C) resemble helminth eggs, with similarities to the intestinal parasites of nematodes and trematode digenetic flukes^32^. The parasites were identified based on their size, shape, and the presence of an operculum in the platyhelminths (in this case open, likely because of dehydration during the fossilization process, see Fig. 1A).

Helminths include parasitic worms that develop as adults in their definitive host, which in many cases, occurs in the digestive tracts of animals. Helminth is a term that encompasses (1) nematodes or roundworms, many of them parasites and (2) flatworms or Platyhelminthes^33^. Platyhelminthes represent the most diverse group of wild vertebrate animal parasites that belong to one of three classes: Trematoda, Monogenoidea or Cestoda^34^. Helminths release a large number of eggs into the environment. This allows them to complete their very complex biological cycle, which may include several intermediate hosts (for Trematodes and Cestodes) until they reach their infective stage for a definitive host, where they will reach sexual maturity and release new eggs inside the host´s faeces once again. A very resistant shell, made up of several layers of various thicknesses, surrounds helminths. This resistant structure is composed of chitin and a lipid covering, which protects external environmental stresses.

## Systematic palaeontology and descriptions

- Phylum Platyhelminthes GEGENBAUR, 1859^35^.
- Class Trematoda RUDOLPHI, 1808^36^.
- Family cf. Opisthorchiidae YAMAGUTI, 1958^37^.

### Description (Fig. 1A)

A trematode egg-like structure was found in the bump-headed lace coprolite MUPA-LH-SnG11 (Fig. 1D). The egg-like structure is not as ovoid as the common shape of the trematode eggs, exhibiting a thicker wall than that of modern trematodes, and with a comparable thickening in the ad-opercular region (Fig. 1A). The size of the fossil parasite structure is 37.9 × 25.1 µm, which is congruent with that of some modern trematode eggs. The egg lacks an operculum, due to the drying of the stool in the fossilization process. This structure is the most sensitive part of a trematode egg, since it must be opened when the miracidium larva are ready to enter into the environment.

### Remarks

There is no modern analogue attributed to this trematode, thereby suggesting a new species, likely related to the digenetic Opisthorchiidae. The eggs of species in the Opisthorchiidae family are generally 21–100 μm in length and 10–120 μm in width^32^. They are generally operculate and yellow/ brown in colour^29^. The eggs of opisthorchiids closely resemble an “amphora”, showing variations in shape among species, which causes identification problems. Ditrich et al.^38^ described these dissimilarities. This variability is present even between eggs of the same species. Therefore, the parasite is ascribed to the cosmopolitan group of digeneans (Fig. 1A).

### Material described (parasite egg)

MUPA-LH-SnG11-Tr (Fig. 1A) from the locality of Las Hoyas (La Huérguina Formation), upper Barremian in age. The specimen is deposited in Museo de Paleontología de Castilla-La Mancha (MUPA) in the Las Hoyas collection.

- Phylum Nematoda COBB, 1932^39^
- Order Ascaridida SKRJABIN & KAROKHIN, 1945^40^

### Description (Fig. 1B)

**Figure 2:**
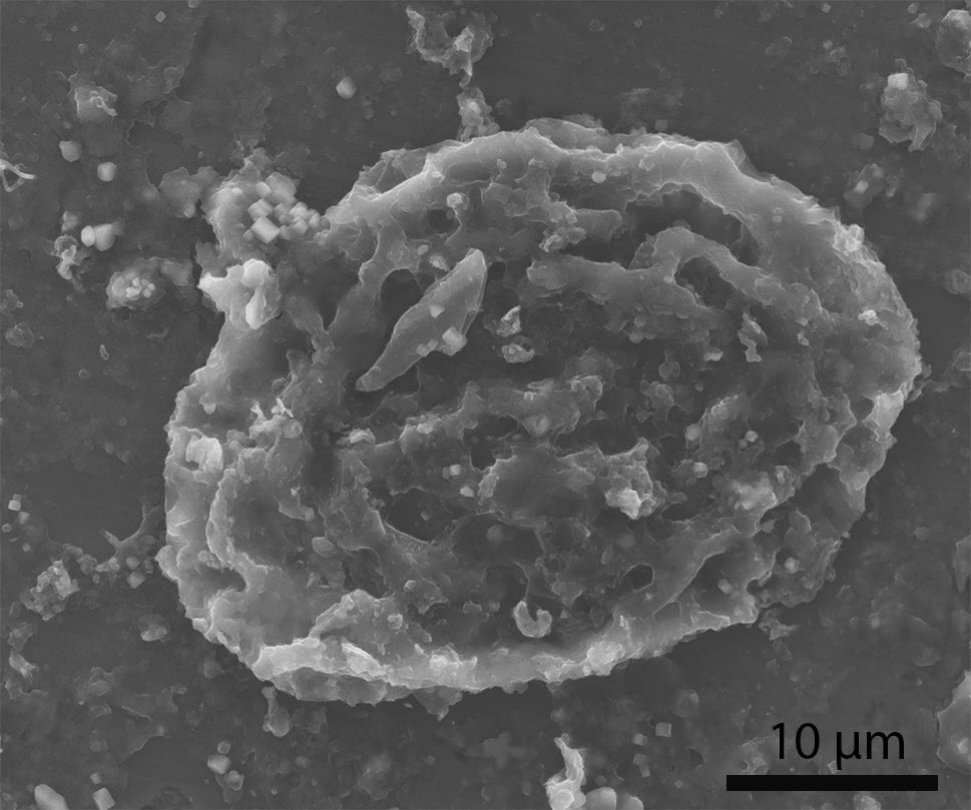
Scanning electron microscope picture (back-scattered electron image) from an Ascaridida egg-like (Nematoda) recovered from the bump-headed lace coprolite MUPA-LH-SnG11-As.

The Ascaridida egg-like structure found in the bump-headed lace coprolite MUPA-LH-SnG11 (Fig. 1D) is not isolated, as a great number of these egg structures were found inside this coprolite (up to 12) (Fig. 2). Their sizes range between 42 × 33 and 64 × 46 µm.

### Remarks

SEM studies suggest that these eggs correspond to ascarids, due to their oval shape and size (42.6 × 34.4 µm). The appearance of the fossilized eggs is similar to that of ascaridids, with a rough fluted cover. Higher magnifications also reveal interconnected ridges, and the surfaces of both the depressions and the ridges appeared textured. It is generally thought that this coating will correspond (at least in the eggs recently emitted by a female) to lipoprotein material that surrounds the chitin that protects the embryo. The external appearance of this egg is similar to that of the eggs of roundworm ascarids^41^.

### Material described (parasite egg)

MUPA-LH-SnG11-As (Fig. 1B) from the locality of Las Hoyas (La Huérguina Formation), upper Barremian in age. The specimen is deposited in the Museo de Paleontología de Castilla-La Mancha (MUPA) in the Las Hoyas collection.

- Family Anisakidae DUJARDIN, 1845^42^

### Description (Fig. 1C)

The other Ascaridida egg-like structure was found in the cylinder coprolite MUPA-LH28719a (Fig. 1E). The size of the fossil parasite structure is 33.7 × 33.2 µm.

### Remarks

The egg wall, the circular shape (quasi-spherical), the smooth outer surface, and the diameter show similarities with the eggs of the *Anisakis* nematode-like *Brevimulticaecum* sp.^43,44^, *Dujardinascaris* sp.^31,44^, and *Terranova* sp^45^.

### Material described (parasite egg)

MUPA-LH28719a-As (Fig. 1C) from the locality of Las Hoyas (La Huérguina Formation), upper Barremian in age. The specimen is deposited in the Museo de Paleontología de Castilla-La Mancha (MUPA) in the Las Hoyas collection.

## Discussion

**Figure 3:**
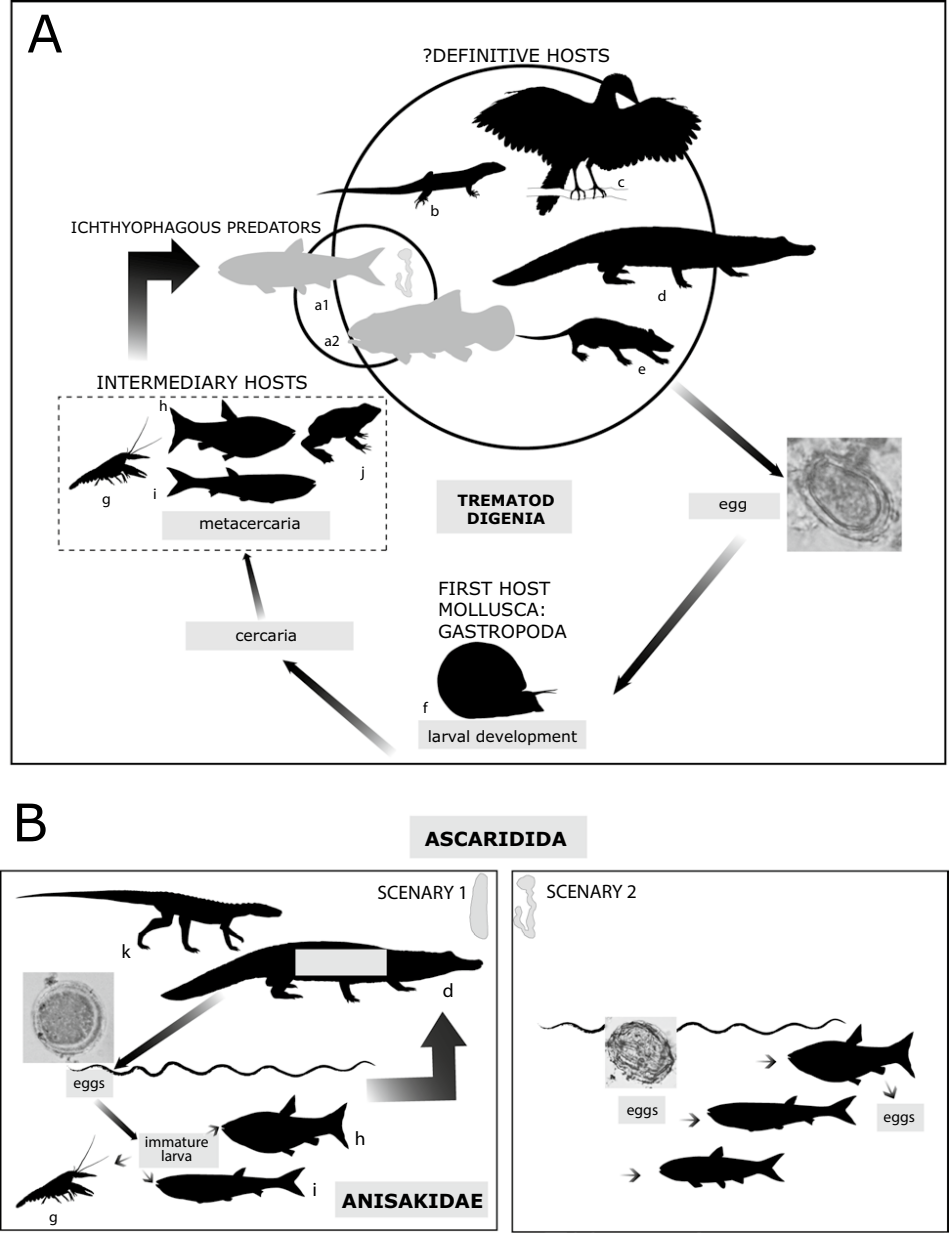
Schematic draw of the hypothetical life-cycle of the parasites found in the lacustrine wetland of Las Hoyas (upper Barremian, Spain). (**A**) Digenea trematode. The grey silhouettes correspond to an adult teleost (a1) or to a young voracious amiiform (a2) that could produce the bump-headed lace coprolite. The putative definitive hosts are (b) the squamate *Meyasaurus*; (c) Enantiornithes; (d) neosuchian; (e) Gobiconodontidae mammals. The first host silhouette is (f) the pulmonated snail *Gyraulus* sp., whereas the intermediary hosts gather (g) decapods; (h) the Gonorynchiformes *Gordichthys* sp., and (i) *Rubiesichthys* sp.; and (j) the Salientia *Gracilibatrachus* sp. (**B**) Ascaridida. Scenario 1 depicts the cycle of Anisakidae, from the adult formed in crocodyliform whose eggs or immature larvae were swallowed by fish and crustaceans. Scenario 2 depicts two possible situations with ascaridid eggs: swallowed or defecated by a fish. Art work by Lara de la Cita (Scientific communicator, Department of Biology, UAM). This work is under a *CC BY-NC-ND* license.

Coprolites are optimal sites for preserving the fossil records of parasites and other digestive and interesting structures (e.g., decapod cuticle, bone remains, muscle, and hair), offering a rich source of information^46,47^. An egg cover and its inner layers provide the necessary resistance to decay, and despite parasitic eggs showing evidence of degradation and deformation, a reasonable identification can be determined. In fact, deformation has been tested under in- vitro experiments processing parasitic eggs with different solutions with laboratory assays (Fig. 1 in^48^). The Las Hoyas coprolites have assisted in dating another event in the evolution of helminths. This discovery constitutes the second report of digenea trematodes and ascaridid eggs from the Early Cretaceous^2,7^. The first report described their presence in archosaur coprolites (crocodile or dinosaur) from the freshwater facies of the Bernissart locality (also Barremian in age^7^). Hence, the Las Hoyas coprolites confirm the presence of ascaridid eggs in crocodylomorph faeces and document a new vertebrate host for the other two parasitic eggs: a fish. The coprolite MUPA-LH-SnG11 documents the role of a fish as an intermediary or as a definitive host in the life cycle of trematodes and ascaridids (Fig. 3A,B), whereas the coprolite MUPA-LH28719a was produced by a reptile (likely a crocodylomorph) as the definitive host (Fig. 3B).

These fossilized parasites also help determine the palaeoecology of this 126–129 Ma freshwater lacustrine inland ecosystem by allowing its comparison with modern tropical analogues. The trophic paths related to the fossilized trematodes and ascaridid eggs are congruent with an interspecific relationship, involving the parasite, the host, and the complex arrays of these ecosystems. In modern freshwater ecosystems, trematode parasites can infect almost all piscivorous vertebrate animals^49^. The life cycles of these parasites include at least three hosts (Fig. 3A). The first larva that hatches from an egg (miracidium) is generally ciliated. This larva swims in the water and penetrates into the first host or is ingested by the first intermediate host (necessarily a mollusc^50^). It is in the first host where a series of larval stages originate (redias and cercarie) by asexual reproduction. This stage is responsible for accessing either the second intermediate host (normally a teleost fish but also crustaceans^51,52^) or encysting within the external environment (depending on the trematode species) to become a metacercaria. Metacercaria is the phase in which the parasite accesses its definitive host and thus where sexual reproduction will take place. The definitive hosts are animals that eat the second intermediate hosts, such as birds, reptiles, and mammals^49,51,53,54^.

For digenea trematodes, such as the fossil described herein, the first intermediate hosts are pulmonated gastropods. Digenea are capable of parasitizing fishes in the liver, gall bladder, bile duct, and rarely in the intestine^32^. Furthermore, some species use crustaceans as intermediate hosts (instead of fishes), where the infective metacercaria phase is formed^32^. The bump-headed lace coprolites, previously attributed to fishes, can be attributed to an adult teleost fish or arguably to a young voracious amiiform^12,25^. An infected teleost fish would have been the definitive host that preyed on a recently infected crustacean (the intermediate host) (Fig. 3A). In the Las Hoyas digenea life cycle, other fish groups bearing metacercaria have been included as intermediary hosts. These fishes correspond to Gonorynchiformes (*Gordichthys* sp. and *Rubiesichthy* sp.), but they are not considered active ichthyophagous species because they feed on larvae and small insects (*Gordichthys* sp.), or they are generalized rams (*Rubiesichthys* sp.). This conclusion was confirmed due to their ecomorphological features^26^. However, their presence in the ecosystem is quite remarkable due to the strong relationship between fish and digenetic trematodes. Most trematode species (except *Pseudogomtiotrema*) parasitize the Ostariophysi teleost Siluriformes^32^, and Gonorynchiformes are close relatives of Siluriformes^55^. The molecular estimate of the Siluriformes node is 133 Ma (mid Early Cretaceous^56^). The oldest known fossil Ostariophysi are Gonorynchiformes *Rubiesichthys* sp. and *Gordichthys* sp., which came from the Berrasian-Barremian Spanish localities of El Montsec and Las Hoyas^26,56^, suggesting that the Las Hoyas ecosystem documents the earliest evidence where digenetic trematodes parasitized the earliest known Ostariophysi.

Similarly, parasites of the phylum Nematoda (Ascaridida) are located in vertebrates living in wetland ecosystems^57,58^. We have also registered nematode body fossils in the Las Hoyas locality^21^. Anisakidae are a family of gastrointestinal nematodes (roundworms) with a complex life cycle. Adult Anisakidae worms lay eggs (oviposition) in the stomach or in the intestine of the definitive host. Then, the eggs are released from the host through faecal matter^58^. The eggs become embryonated in the water, and the infective L3 larvae hatch from eggs as free-swimming larvae that are ingested by crustaceans or fishes^44,59^. These animals could be ingested directly by the definitive host or by a new fish, in the latter case remaining as paratenic hosts until reaching the definitive host by ingestion. Then, the larvae develop into adults in the stomach or in the intestine of the definitive host. Modern *Brevimulticaecum* sp. and *Dujardinascaris* sp. anisakids are associated with crocodyloids and alligatoroids as definitive hosts^31,43,44^.

The shape, size, absence of inclusions, and parasitic content of the cylinder coprolite MUPA-LH28719a are fully congruent with the presence of *Ascarid* sp. eggs, which are frequently found in Mesozoic crocodyliform coprolites^6,60^, as well as in extant members of the Crocodylia clade^31,43,44^. Thus, the anisakid lifecycle started with an infected crocodyliform (Fig. 3B, scenario 1), in whose alimentary tract the adult worm lived. Then, the crocodyliform produced a faecal mass with a large number of eggs inside, and the faecal mass was deposited into the water. This scenario involves two different crocodylomorph groups, both present in the Las Hoyas assemblage: a basal Gobiosuchidae represented by *Cassissuchus* sp., and a member of the stem Crocodylia in which modern groups phylogenetically belong^61^. In addition, for the ascaridids retrieved in the fish faecal mass (MUPA-LH-SnG11), a second scenario has been suggested (Fig. 3B). The fish would have (i) ingested the egg with the plankton and emitted it via faeces without being altered by intestinal transit, or (ii) the parasite egg could correspond to an egg emitted by a female adult that parasitized the fish. In both cases, possible embryonic development in the egg could not be determined since only the external egg surface was observed. In the fish scenario, Gonorynchiformes and other teleosts are considered to have been coprolite producers due to their preferred non-ichthyophagous trophic habits^26^.

The highly complex life cycles of digenetic trematodes and ascaridids encompass a variety of invertebrate and vertebrate groups (i.e., mammals, birds, reptiles, fishes, snails, bivalves, crustaceans, annelids, and insects; see^62^ for trematodes). The lacustrine ecosystem of Las Hoyas contains many of these groups that are currently involved in parasitic cycles: pulmonated gastropods (*Gyraulus* sp. and *Prophysa* sp.), unionid bivalves^63^, heteroptera Belostomatidae^64^, worm animals attributed to several Oligochaeta annelids^21^, and crab and shrimp crustaceans^65^. Furthermore, by probing for parasites in coprolites, we tested the relevance of fishes in the lifecycles of trematodes and ascaridids. The evidence provided by these fossilized parasite eggs helps us to obtain a clearer picture of this Early Cretaceous wetland ecosystem, and it will be useful for further comparisons with other relevant Peritethys lacustrine ecosystems, such as Araripe in Brazil^66^ or El Montsec in Catalonia^67^.

## Methods

A total of twenty-nine coprolites were used in this study (see Supplementary Information). The coprolite matrix was extracted using a punch, and then slightly ground using an agate mortar and pestle. All tools used during the extraction and preparation of the coprolites were carefully cleaned with 70% ethanol, and then rinsed three consecutive times using ultrafilter (0.45 µm) distillate water between the samples to avoid cross-contamination.

The samples were prepared in the laboratory of the Molecular Parasitology Department at the Universidad de Granada, Spain, following the modified protocol of Ferreira et al.^68^. The crushed coprolites were dissociated in an acid buffer of glycine 0.1 M, ethylenediaminetetraacetic acid (EDTA 0.15 M) pH3 to remove calcium and/or magnesium carbonate. The samples were processed over two weeks in sterile conical centrifuge tubes, and shaken manually twice per day. Then, the samples were centrifuged at 1500 r.p.m for 10 min to concentrate the pellet with the insoluble material. Then, the supernatant was extracted following the modified protocol of Ritchie^69^. The samples were washed in distilled water to minimize the acid reaction on the recovered residues, and then centrifuged at 1500 r.p.m for 10 min to concentrate the pellet. Up to ninety-nine slices were prepared and studied at Centro de Instrumentación Científica (CIC) (University of Granada, Spain) under an optical microscope (Olympus BX51) at × 200 and × 400 final magnifications, and pictures were taken with an Olympus DP50 digital camera (effective resolution: 2.776 × 2.074 pixels) using the photographic system PM10SP1. The sample treated for the scanning electron microscopy study was dehydrated with an alcohol series and then transferred to acetone, dried under a critical point, placed on a stub coated with gold, and examined under a scanning electron microscope (ESEM) FEI, mod. QuemScan650F, at the CIC. The image was taken in backscattered electron mode. The photographed structures were measured using ImageJ software^70^.

## Supplementary information

Supplementary Information.
