# Supplementary material for: Helminth eggs from early cretaceous faeces
Source: Sci Rep. 2020 Oct 30;10:18747. doi: 10.1038/s41598-020-75757-4 (PMC7599231; doi:10.1038/s41598-020-75757-4)
Supplement: Supplementary file 1 — Supplementary Information. [file 41598_2020_75757_MOESM1_ESM.pdf]

Helminth eggs from Early Cretaceous faeces

Sandra Barrios-de Pedro[1*], Antonio Osuna[2,3] & Ángela D. Buscalioni[1]

[1] Unidad de Paleontología and Centro para la Integración en Paleobiología (CIPb), Universidad Autónoma de Madrid. Departamento de Biología, Edificio de Biología, Calle Darwin 2, 28049 Cantoblanco, Madrid, SPAIN. sbarriosdepedro@gmail.com; angela.delgado@uam.es.

[2] Departamento de Parasitología, Universidad de Granada. Facultad de Ciencias, Avenida de la Fuente Nueva s/n, 18071 Granada, Granada, SPAIN. aosuna@ugr.es.

[3] Instituto Universitario de Biotecnología. Edificio Fray Luis de Granada, Calle Ramón y Cajal 4, 18071 Granada, Granada, SPAIN.

(*) Corresponding author.

Supplementary information

Morphotype	Specimens
**Bump-headed lace**	MUPA-SnG11; MUPA-LH-LI15-031.
**Circular**	MUPA-LH-LI15-012.
**Cylinder**	MUPA-LH28719a; MUPA-LH22141; MUPA-LH21055; MUPA-LH17075; MUPA-LH9534; MUPA-LH15900; MUPA-LH21067; MUPA-LH-LI15-026.
**Elongated**	MUPA-LH-LI15-001; MUPA-LH-LI15-002; MUPA-LH-LI15-014_2.
**Fir-tree**	MUPA-LH-LI15-021.
**Irregular**	MUPA-LH30254; MUPA-LH28775; MUPA-LH-LI15-006; MUPA-LH-LI15-027.
**Spiral**	MUPA-LH-LI15-032.
**Straight lace**	MUPA-LH-LI15-005; MUPA-LH-LI15-015; MUPA-LH-LI15-035.
**Thin lace**	MUPA-LH-LI15-007; MUPA-LH-LI15-014_1; MUPA-LH-LI15-017; MUPA-LH-LI15-018.
**Broken**	MUPA-LH12028; MUPA-LH9195.

Supplementary. The 29 coprolites studied and their correspond morphotype, following the classification proposed by Barrios-de Pedro et al. [24].
